# Retraction: Long non-coding RNA ANRIL promotes the invasion and metastasis of thyroid cancer cells through TGF-β/Smad signaling pathway

**DOI:** 10.18632/oncotarget.28821

**Published:** 2025-12-31

**Authors:** Jian-Jie Zhao, Shuai Hao, Ling-Li Wang, Chun-Yan Hu, Shu Zhang, Ling-Ji Guo, Gang Zhang, Bo Gao, Yan Jiang, Wu-Guo Tian, Dong-Lin Luo

**Affiliations:** ^1^Department of Breast, Thyroid, and Vascular Surgery, Research Institute of Surgery, Daping Hospital, Third Military Medical University, Chongqing, 400042, P. R. China

**This article has been retracted:** Oncotarget has concluded its investigation of this paper. Multiple instances of internal and external duplications and overlaps of Transwell assy images were discovered. These include:

Internal Duplications:

Figure 5, TPC-1, panel A is a duplicate of SW579 panels A and B, and correspondingly SW579 panels A and B are duplicates too.

Figure 5, TPC-1, panel E overlaps with SW579 panel D.

Figure 6, TPC-1 panel A overlaps with panel B.

External Duplications:

Figures 5 and 6 contain panels that overlap with earlier published figures 7 and 12 in already retracted article [1] and Figure 8D in article [2]. Concurrently published papers [3] and [4] also shred images from Figures 5 and 6. And multiple panels from Figures 5 and 6 were found in later published unrelated articles [5–8].

The authors and their institution have been contacted, but neither has responded. Therefore, the Editorial decision has been made to retract the paper.

Original article: Oncotarget. 2016; 7:57903–57918. 57903-57918. https://doi.org/10.18632/oncotarget.11087
